# Evaluating the Utilization of Ethylenediaminetetraacetic Acid as a Treatment Supplement for Gliomas

**DOI:** 10.7759/cureus.31617

**Published:** 2022-11-17

**Authors:** Muhammad S Ghauri, Akshay J Reddy, Ethan Tabaie, Leo Issagholian, Telak Brahmbhatt, Yui Seo, Allen Dang, Neel Nawathey, Alex Bachir, Rakesh Patel

**Affiliations:** 1 Neurosurgery, California University of Science and Medicine, Colton, USA; 2 Medicine, California University of Science and Medicine, Colton, USA; 3 Neurobiology, Physiology and Behavior, University of California, Davis, Davis, USA; 4 Health Sciences, California Northstate University, Rancho Cordova, USA; 5 Anaesthesia, California Northstate University College of Medicine, Elk Grove, USA; 6 Medicine, Geisinger Commonwealth School of Medicine, Scranton, USA; 7 Internal Medicine, East Tennessee State University Quillen College of Medicine, Johnson City, USA

**Keywords:** cell lysis, cancer, chelation, neuroimaging, glioma, edta

## Abstract

Gliomas are the most common primary tumors of the nervous system, accounting for approximately 81% of brain tumors. The primary treatment for these primary brain tumors, especially those of high grade, is surgical resection with subsequent therapy such as targeted radiotherapy, chemotherapy, or supportive care. In an effort to devise nuanced ways to treat gliomas, studies have examined various chemical agents to expand therapeutic avenues for gliomas. In this study, we investigate the applications of ethylenediaminetetraacetic acid (EDTA) in the investigation and treatment of gliomas. Searches were conducted on PubMed to find studies about the use of EDTA in the treatment of glioma. We identified 36 studies that had the information needed for analysis. We collected information on the dosage of EDTA, the agent that EDTA was complexed with, the route of administration, the outcome of the EDTA usage, and the type of glioma cells that were involved. In addition, a one-way analysis of variance was performed to identify any relationships between the effect of cell type, study purpose, and year published on dosage. We identified 36 articles that met our inclusion criteria. In-vitro studies utilized EDTA in various complexes to evaluate cellular viability, including proliferation and toxicity, intracellular enzyme kinetics, and intercellular interactions such as chelation and cellular aggregation. In-vivo studies predominantly utilized the versatile nature of EDTA as a tracer for imaging studies involved in diagnostics and identifying recurrent tumor growth and localization in human patients. Our statistical analysis failed to identify any significant relationships between cell type, study purpose, and publication year on EDTA dosage. We identified a variety of uses for EDTA in the investigation hopefully providing physicians with information regarding the context and applications of EDTA to assist in exploring new treatment options for glioma patients.

## Introduction and background

Gliomas are the most common primary tumors of the nervous system. They originate from glial cells or glial precursors. They can be classified histologically based on their resemblance to other native cells regardless of the cell of origin or based on biochemical analysis [1]. While the etiology of tumorigenesis is unclear, prevailing theories include the cancer stem cell model, which suggests that specific glial stem cells are predisposed to tumorigenesis. The clonal model suggests that competitive mutations enhance the survival of a cell lineage leading to the accumulation of these competitive mutations and, ultimately, tumorigenesis and random deleterious mutations which occur at key genes including but not limited to RAS, Kit, p53, NF1, PTEN, growth factor receptors, isocitrate dehydrogenase (IDH), and more as they are uncovered [2,3]. Gliomas account for 81% of brain tumors [4]. In an analysis of 244,808 patients from the Central Brain Tumor Registry of the United States (CBTRUS), the incidence and types of diagnosed gliomas were reported as glioblastomas (61.5%), oligodendroglia, tumors (10.7%), ependymomas (3.6%), other gliomas (5.4%), and non-glioblastoma astrocytomas (18.8%) [4]. The most common malignancy was glioblastoma (45.2%), with its incidence increasing with age to a peak in the eighth decade of life [5,6]. Glioblastomas have a poor prognosis, with a five-year survival rate estimated at 2.7-8.9% [7,8]. Malignant, grade IV glioblastomas are termed glioblastoma multiforme (GBM), and contributing to the low survival rates of GBM are high rates of infiltration, heterogeneity of tumors which complicates T-cell responses and targeted therapy, areas of blood-brain barrier (BBB) salvage which prevents chemotherapeutic against from reaching tumor cells, and immunosuppressant tumor environment [6]. Evaluation and delineation of proper tumor borders, the integrity of the BBB, and the distribution of glioma can help with appropriate treatment. Targeted therapy of gliomas can vary based on classification. The primary treatment for primary brain tumors, especially those of high grade, is resection with subsequent radio or chemotherapy [9]. Almost half of the low-grade gliomas may not receive surgical resection [10]. Alternative or adjunct therapies can include targeted radiotherapy, chemotherapy, or supportive care, with the greatest median survival reported from combined radiotherapy and carmustine wafer [9]. Targeted radiotherapy is a multi-step process. Initial imaging with MRI is done to delineate the borders of the remaining mass or for a mass that could not be resected. Radiation dosage is based on the likelihood of recurrence [11]. A decreased time to treatment is associated with a greater overall survival rate in gliomas [12]. Varying monoclonal antibodies can be utilized based on histological and genetic characteristics of tumor cell lines [3]. Interestingly, targeted isocitrate dehydrogenase (IDH) therapy has been applied for glioblastomas, including vaccines and antibodies with better prognosis following resection [3,13]. There are multiple phase II trials investigating the use of nivolumab in IDH-mutated malignancies [14]. A concern, however, is that repeated non-surgical treatments offer diminishing returns with decreased progression-free survival rates in repeated treatments of low-grade gliomas [10]. The emergence of new therapies can help ameliorate the concerns of treatment-resistant gliomas. In an effort to devise nuanced ways to treat glioma, studies have evaluated different chemical agents to understand treatment avenues better. In this study, we investigate the applications of ethylenediaminetetraacetic acid (EDTA) in the diagnosis, research, and treatment of gliomas. EDTA is most commonly used as a chelating agent in the treatment of lead poisoning [7-13]. The objective of this study is to provide physicians with information regarding the context and applications of EDTA to assist in exploiting new treatment options for glioma patients.

## Review

Methodology

**Figure 1:**
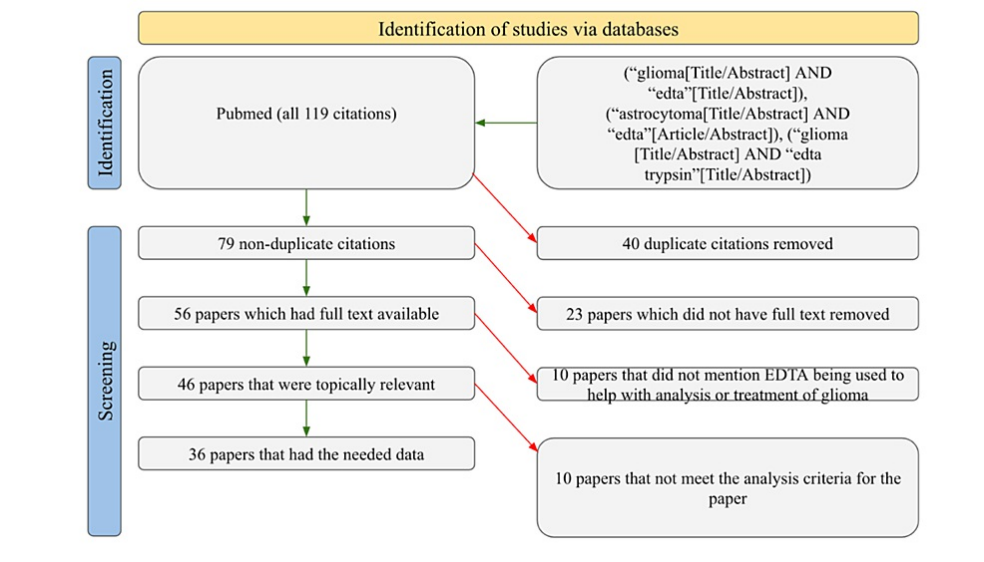
PRISMA diagram illustrating the article filtration process. PRISMA: Preferred Reporting Items for Systematic Reviews and Meta-Analyses

We performed literature searches on PubMed to find studies about the use of EDTA in the treatment of gliomas. All timeframes were investigated. This resulted in a total of 119 papers, of which 79 were unique, 56 had full text accessible, 46 were relevant to our scope, and only 36 included the necessary data for analysis. In investigating these 36 papers, the authors gathered information on the dose of EDTA, the EDTA complex that was used, the mode of administration, the outcome of EDTA use, and the kind of glioma cells involved. Studies that lacked sufficient data to overlap with at least two of the aforementioned categories were eliminated. No articles meeting the criteria were omitted, and each article was evaluated by each of the authors, offering a robust analysis of respective papers. Figure 1 illustrates our filtering procedure, according to the Preferred Reporting Items for Systematic Reviews and Meta-Analyses (PRISMA) guidelines, used in this study. A one-way analysis of variance (ANOVA) was performed to identify any relationships between the effect of cell type, study purpose, and year published on dosage. All statistical analyses were performed using SPSS statistics software version 28.0.1.0 (IBM Corp., Armonk, NA, USA).

Cellular proliferation and toxicity

We identified 36 articles that met our inclusion criteria. This information is displayed in Table 1 [15-50]. In-vitro studies utilized EDTA in various complexes to evaluate cellular viability, including proliferation and toxicity in glioma cells. It should be noted that C6 glioma cells were the most commonly reported cell line of glioma cells that were utilized among the articles analyzed in the review. EDTA has been shown to reduce the proliferative capacity of certain glioma/glioblastoma cell lines. When complexed with 4Na or Ca, EDTA exhibited non-toxic anti-proliferative effects in the presence of soybean trypsin inhibitor (c6 glioma cells) and Tenascin C (u87 glioma cells) [15,19]. Similarly, 0.4 µM of human serum albumin EDTA improved endocytosis and conversion of vanadium into an anti-proliferative agent in CNS-1 glioma cells [24]. EDTA toxicity has been evaluated in c6 cells and 1321N1 human astrocytoma cells at 0.002 µM to 4 mM doses. Following GlioN6,2'-O-dibutyryl-cAMP (DBcAMP) pulses which increase cAMP, leading to morphological and inflammatory changes, EDTA was found to be non-toxic [18]. EDTA has been used in a variety of pre-clinical and clinical applications in the context of glioma. The most common complex utilized was ^68^[Ga]EDTA at a dose of 5-10 MCi.

**Table 1:** Current applications of EDTA in treatments for gliomas. EDTA: ethylenediaminetetraacetic acid; HSV-1: herpes simplex virus 1; BBB: blood-brain barrier; HSA: human serum albumin; TEER: transepithelial/transendothelial electrical resistance; PET: positron emission tomography; GAG: glycosaminoglycan; PDT: photodynamic therapy; MRI: magnetic resonance imaging; GBM: glioblastoma multiforme; ELISA: enzyme-linked immunosorbent assay; WHO: World Health Organization

Author (year)	Dosage	Complex	Route of administration	Outcome	Type of glioma
Amano et al. (1996) [15]	0.53%	Trypsin EDTA-4Na	In vitro	Soybean trypsin inhibitor inhibited glioma cell proliferation	C6 glioma cells
Ando and Arai (1980) [16]	0.02%	Trypsin EDTA-4Na	In vitro	Trypsin increased cellular synthesis of HSV-1 antigens	C6 glioma cells
Bergström et al. (1983) [17]	N/A	^68^[Ga]EDTA	In vivo	^68^[Ga]EDTA was a definitive improvement for BBB integrity assessment	Anaplastic astrocytoma
Black et al. (1997) [18]	10–300 ng/kg (RMP-7) 5–10 mCi (Ga)	Recombinant bradykinin (RMP-7) and ^68^[Ga]EDTA	Intracarotid infusion	RMP-7 mediated increase in BBB permeability led to 50% decrease in tumor size in 3/9 patients	GBM/anaplastic astrocytoma
Brellier et al. (2011) [19]	10 mM	Calcium	In vitro	EDTA decreases Tenascin C-mediated migration of U87 glioma cells	Oligodendrogliomas, astrocytomas, glioblastomas
Brismar and Collins (1989) [20]	10 mM	Magnesium free	In vitro	Glial cell K+ membrane permeability is important for ion homeostasis in the brain	Tp-276MG/301MG/378MG/483MG, U-251MG (human malignant gliomas)
Reichard-Brown and Akeson (1983) [21]	0.2% EDTA	EDTA-4NA	In vitro	No difference between adhesion when comparing EDTA to trypsin only. Can be used for removing cells from culture vessels	C6
Lo Cicero et al. (2012) [22]	10 mM	No complex	In vitro	EDTA inhibited metalloprotease activity	G26/24 oligodendroglioma cells and synovial fibroblasts
Constantinovici (1972) [23]	1 mL of 10% EDTA – 10 mCi of ^113m^In	^113m^In EDTA	In vitro	EDTA chelation improves tumor uptake and tumor classification scintiphotographic	Oligodendrogliomas
Cooper et al. (2021) [24]	0.37 µmol	HSA-EDTA	In vitro	HSA-EDTA administration improves endocytosis and vanadium conversion into an anti-proliferative agent	CNS-1 glioma cells
Dall’Igna et al. (2013) [25]	0.05%	Trypsin-EDTA	In vivo	Trypsinizing cells for detachment	C6 glioma cells
Deininger et al. (1998) [26]	0–0.25 M	N/A	In vitro	EDTA reduces AP-linked antibody crossreactivity in immunohistochemical staining	Astrocytomas (human GBM)
Denora et al. (2017) [27]	1 mM	Na_2_EDTA	In vitro	F-Dopa EDTA lactate-based formulations were better tolerated in rats	Neuronal SH-SY5Y cell lines
Easton and Abbott (2002) [28]	2 mM	Trypsin-EDTA	In vitro	EDTA treatment abolished the bradykinin plateaued TEER	C6 glioma cells
Ericson et al. (1985) [29]	N/A	^68^[Ga]EDTA	In vivo	EDTA tracers in PET is sensitive in detecting BBB barrier ruptures	Anaplastic astrocytomas
Fujikawa et al. (2015) [30]	0–2 mM	N/A	In vitro	Use of EDTA prevents MTT reduction in the presence of ZnCl	Astrocytic C6 glioma cells
Glimelius et al. (1978) [31]	0.5 mM	No complex	In vitro	Trypsin-EDTA decreased incorporation of 35S-glycosaminoglycans in glioma cultures	Human-derived U-787 CG malignant glioma cell line
Glimelius et al. (1979) [32]	0.02%	Trypsin	In vitro	EDTA can be used to detach cells from culture. Produces more rounded cells. Solubilized GAG	Human-derived U-787 CG malignant glioma cell line
Guo et al. (2015) [33]	5 mM	No complex	In vivo	EDTA exposure to the functionalized quantum dots demonstrated excellent radiographic stability	U87MG glioblastoma xenograft models
Hong et al. (2009) [34]	0.05 %	EDTA-trypsin	In vitro	EDTA can be used to detach cells from culture following PDT treatment	U251n and U87 glioma cell lines
Hung and Lu (2001) [35]	1 mM	No complex	In vitro	Cell lysis	C6 glioma
Ilsen et al. (1984) [36]	5–8 mCi	^68^[Ga]EDTA	In vivo	EDTA tracers in PET are sensitive in diagnosing brain tumors recurrent tumor growth. PET EDTA shows extravasation of radionuclide and information on the the BBB	Glioblastoma, mixed glioma, astrocytoma
Johnström et al. (1987) [37]	300 MB_q_	^68^[Ga]EDTA	In vivo	Preliminary PET studies indicate [1-^11^C]methionione is superior to ^68^[Ga]EDTA in detecting BBB disruption detection	Astrocytoma
Jung et al. (1999) [38]	10 mM	^68^[Ga]EDTA	In vitro	Protease inhibitor	U87 MG astrocytoma
Kagaya et al. (1995) [39]	1 mM	No complex	In vitro	EDTA was utilized as a chelator to remove Ca^2+ ^ions 5-HT-induced cGMP production	C6 glioma
Krushelnychy et al. (1991) [40]	N/A	Gd-EDTA	In vivo	Gd-EDTA enhanced MRI imaging for tumor localization in GBM xenogratft models	Human glioma-derived D-54 MG (GBM)
Leis et al. (1982) [41]	5 mM	No complex	In vitro	EDTA failed to inhibit tyrosine protein phosphatase activity	Astrocytoma
Lilja et al. (1985) [42]	5 mCi	^68^[Ga]EDTA	In vivo	L-methyl-^11^C-methionine is an alternative to ^68^[Ga]EDTA for low-grade astrocytoma imaging	Astrocytoma
Lilja et al. (1989) [43]	N/A	^68^[Ga]EDTA	In vivo	^68^[Ga]EDTA was used to evaluate the integrity of the BBB to estimate changes in local blood volume and determine residual vs. recurrent glioma	Astrocytoma
Mair et al. (2021) [44]	N/A	No complex	In vivo	EDTA plasma was used for spD-L1 ELISA detection during bevacizumab treatment	Recurrent WHO grade II-IV glioblastomas
Mair et al. (2020) [45]	N/A	No complex	In vivo	EDTA plasma was used for spD-L1 ELISA detection during bevacizumab treatment	Glioblastoma and lower-grade glioma
Mead and Pentreath (1998) [46]	0.002 µM to 4 mM	Trypsin	In vitro	EDTA was tested as a toxicity agent for glioma cells and was found to not be significantly toxic following dBcAMP pulse	C6 glioma and 1321N1 human astrocytoma cell lines
Miller et al. (1976) [47]	10 mM	Ammonium sulfate	In vitro	EDTA was used to evaluate ATPase stimulation of myosin extracted from	C6 glioma N2A neuroblastoma
Ricklefs et al. (2019) [48]	N/A	N/A	In vitro and in vivo (patient plasma)	EDTA was used as anticoagulant for all plasma samples to detect extracellular vesicles in malignant brain tumors	GS-5, GS-8, GS-57, GS-60, GS-74, BT112, NCH644, GSC168, GSC233, GSC407
Wang et al. (2010) [49]	50–100 µM	Na_2_EDTA	In Vitro	EDTA reverses aggregation/binding of Zn^2+^-β-amyloid peptide	SHG-44 human glioma cell
Wu and Waxman (2015) [50]	2 mM	N/A	In vitro	EDTA can be used as an affected resuspension medium	GL261 glioma cells

Enzyme kinetics

In addition, EDTA has been used to study intracellular enzyme kinetics within glioma cells and intercellular interactions such as chelation and cellular aggregation. EDTA has demonstrated a role in protease inhibition when complexed with^ 68^[Ga] in U87MG astrocytoma cells [38], as well as metalloproteinase inhibition in G26/24 oligodendroglioma cells, which regulates regional cellular invasion [20]. Chelation studies revealed that 1 mM of EDTA could attenuate 5-HT-induced cGMP production in c6 glioma cells, leading to regulatory changes in glial signaling systems [21]. EDTA has also been utilized to study cellular aggregation in malignant brain tumor samples as an anticoagulant to detect extracellular vesicles via flow cytometry [22]. Similarly, 50-100 µM of Na_2_EDTA has been shown to reverse the aggregation of Zn^2+^-β-amyloid peptide in SHG human glioma and glioblastoma-derived stem cells, modulating neurodegenerative pathologies [27].

Cell lysis

EDTA was complexed with several different compounds to achieve both cell lysis and especially detachment. Trypsin was often used in complexes with EDTA to enhance trypsinization, the process by which trypsin, a protease that breaks down adherents, prevents the attachment of cells to their surrounding structures [24-27]. Trypsin-EDTA seemed effective as a method of cell detachment in vitro [26,27]. However, when separated, EDTA and trypsin seemed to have no difference in adhesion when utilizing a solution of 0.2% EDTA in vitro [24]. One in-vivo experiment also involved trypsin-EDTA as a method of cellular detachment by utilizing a solution of 0.05% trypsin-EDTA [25]. Several studies have effectively demonstrated the utility of trypsin-EDTA in cell detachment, where cells must be removed and transported from their attachment sites [24-27]. EDTA without a complex can also be used as a lysis buffer, as was described in two in-vitro studies involving C6 glioma cell lines [21,28]. EDTA is able to chelate divalent cations, which serve as cofactors for many essential enzymes, thereby serving as an effective lysis buffer. It can also be used in protein extraction due to similar properties [29-32]. In two studies, uncomplexed EDTA was able to enhance the specificity of an enzyme-linked immunosorbent assay through its ability to prevent divalent cations from contaminating the experiment [30,31]. In one study, EDTA showed inhibition of alkaline phosphatase through its chelation of essential divalent cation cofactor [29]. As a result, any future experiments requiring targeted control of enzyme function in glioma cells could use EDTA if that enzyme requires a divalent cation to function.

Imaging

Many of the in-vitro findings of EDTA usage in gliomas have led to its translation into clinical applications. In-vivo studies using EDTA solutions predominantly leveraged its versatile composition and relatively low permeability in the BBB and other cortical areas under normal physiologic conditions [36]. This enabled it to serve as an effective tracer in imaging studies. ^68^[Ga]EDTA was used in four different positron emission tomography studies to evaluate BBB permeability in 65 human glioma patients (GBM, mixed glioma, anaplastic astrocytoma) [36-39]. ^68^[Ga]EDTA was also used concomitantly with RMP-7 to increase BBB permeability, leading to a 50% decrease in tumor size in three out of nine adult GBM patients. ^68^[Ga]EDTA tracers have been shown to effectively diagnose various glioma tumor subtypes in human patients. In addition, a few studies note its ability to identify recurrent tumor growth and localization pre-clinically in xenograft models, as well as in human patients [36-38]. A one-way ANOVA was performed to identify any relationships between the effect of cell type, study purpose, and year published on dosage. Our results revealed that there was no statistically significant difference in dosage between cell type (F(11,13) = 0.379, p = 0.467), study purpose (F(11,13) = 0.430, p = 0.925), and the year published (F(11,13) = 1.621, p = 0.214). Overall, these findings indicate that EDTA dosage is highly variable depending on specific study parameters and does not display any significant relationships. This corroborates the potential utility of EDTA in various capacities, including investigation in the context of glioma.

**Table 2:** One-way analysis of variance results evaluating effects on the mean dosage.

Variables	df	Mean square	F	Significance	95% confidence interval	
Year published	13	2.78	1.621	0.214	0	2.248
Cell type	13	0.802	0.379	0.925	-1.904	-1.139
Study purpose	13	2.801	0.430	0.467	0	0.488

Functionality

EDTA is an acid that is approved for use as a medication for heavy metal toxicity in patients [39]. EDTA functions as a chelating agent used to remove heavy metals, such as lead and mercury, from the bloodstream of patients [39]. The mechanism by which this occurs is that EDTA can form either four or six bonds with metal ions, forming chelates that can remove heavy metals from the bloodstream [40]. While chelation therapy is necessary in heavy metal toxicity cases, it can also have unintended side effects, such as EDTA-associated hypocalcemia, especially when used for pediatric treatment [41]. EDTA also inhibits metalloproteases as it chelates divalent cations necessary for activity [39]. EDTA has been shown to also enhance the cytotoxic effects of various compounds in a dose-dependent manner [42]. Overall, EDTA has demonstrated great utility in various applications, but further research should be conducted to understand its vast effects fully. In this investigation, we were able to corroborate information regarding how EDTA was used in the treatment and analysis of patients with glioma. Through the analysis of over 30 different articles, we were able to gain insight into how this cheating agent could be used to elucidate various properties of this cancer [15-38,43-54]. One of the main benefits of working with EDTA was its use as a radioactive image tracer, in the form of ^68^[Ga]EDTA, allowing researchers to obtain a significantly clearer view of patient pathologies to diagnose better, classify, and identify recurrent growth of this disease [34]. Because the current standard of treatment does not incorporate ^68^[Ga]EDTA as an imaging tracer for glioma patients, incorporating this standard is something that should be considered when looking toward improving patient-centered outcomes. Another advantage of EDTA in the treatment of glioma is the fact that this chelator can be utilized to enhance the function of cytotoxic agents that serve to directly reduce the size of the tumors that this condition propagates [23]. This information should be taken into consideration when clinicians are evaluating the numerous options for glioma patients, as reducing tumor sizes improves candidacy for surgical resection and could significantly improve the treatment outcomes and quality of life [31]. Although the investigation was conducted in a meticulous manner, there were some limitations in this investigation. For instance, several studies discussed using EDTA as a buffer reagent for cell lysis and detachment for further analysis. However, these studies did not investigate or even discuss whether using EDTA as a detachment agent may have had confounding effects that could skew the results that were presented [29-31,49]. Future investigations using in-vitro glioma cultures could investigate whether there is a statistical significance in the outcomes when EDTA is used instead of an alternative buffer or cell lysis agent. Another limitation of this investigation was the fact that there were very few in-vivo studies that were collected with a significant sample size or even reported a sample size [15-38,42-54]. Finally, we were unable to include in-vivo studies in our ANOVA analyses because EDTA dosages were administered in units of radioactivity. We omitted these parameters as they could have skewed the reliability of the results that were reported. Therefore, future investigations on this issue should attempt to obtain a larger sample size of glioma patients to confirm the validity and reliability of these conclusions.

## Conclusions

We compiled and filtered through several studies to create a review that discussed information about the relationship between EDTA, glioma treatment, and glioma cell analysis. When used to treat patients with glioma, EDTA was shown to promote cytotoxic effects of specific glioma cells, which resulted in reduced tumor size and a decrease in associated symptoms. In this review, we found that EDTA was utilized as a radiographic tracer to enhance the quality of glioma tumor images that were captured by imaging modalities. EDTA was also successfully used in numerous investigations of glioma cells as a cell lysis agent and a protease inhibitor. Within in-vitro studies, we found that there was no significant difference in the concentration of EDTA that was being used to analyze glioma cells, despite the different purposes and tests that utilized EDTA. This could potentially indicate that EDTA may be used in various study capacities with large therapeutic windows for dosing. However, further investigations are needed to validate these findings.
